# Rethinking Intelligence Quotient Exclusion Criteria Practices in the Study of Attention Deficit Hyperactivity Disorder

**DOI:** 10.3389/fpsyg.2016.00794

**Published:** 2016-05-31

**Authors:** Genevieve B. Mackenzie, Elif Wonders

**Affiliations:** ^1^Attention Deficit Hyperactivity Disorder and Literacy Laboratory, Ontario Institute for Studies in Education, University of TorontoToronto, ON, Canada; ^2^The Connections Laboratory, Department of Education and Counselling Psychology, McGill UniversityMontreal, QC, Canada

**Keywords:** attention deficit/hyperactivity disorder, intelligence quotient, exclusion criteria, inclusion criteria, mild intellectual impairment

## Abstract

Attention deficit hyperactivity disorder (ADHD) is associated with lower than average intelligence quotient (IQ) scores. However, research done on this disorder often excludes participants based on lower than average IQ’s (i.e., between 70 and 85). The purpose of this paper is to alert researchers to the consequences of excluding participants based on IQ’s within this range and to highlight the importance of providing a clear rationale when choosing to exclude participants based on IQ. Next, we offer recommendations for researching ADHD and their relative benefits and drawbacks of these approaches. Overall this paper emphasizes that including participants who have lower than average IQ in research on ADHD may promote a more realistic understanding of the condition and in turn improve our ability to treat it.

## Introduction

Attention deficit hyperactivity disorder (ADHD) is characterized by age inappropriate levels of inattention and/or hyperactivity and impulsivity (American Psychiatric Association [APA], 2013). It affects approximately 4–10% of school aged children frequently persisting into adolescence and adulthood (Barkley, 1997a; Skounti et al., 2007). ADHD is often also associated with lower intelligence quotient (IQ; e.g., Crosbie and Schachar, 2001). For instance, Frazier et al. (2004) reported in their meta-analysis that in comparison to individuals without ADHD, individuals with ADHD score an average of 9 points lower on most commercial IQ tests. In this paper we aim to address the validity of the role IQ plays in the exclusion of potential participants in studies centering on ADHD. First, we describe ADHD IQ exclusionary criteria. Next, we outline the consequences of the exclusion of potential participants with lower IQ. Finally, we discuss recommendations for research studying ADHD when also taking IQ into account.

## IQ Exclusionary Criteria

It is common practice to exclude participants with and without ADHD who have IQ’s that are one to two standard deviations below average (i.e., 85–70). However, excluding participants who are above one or two standard deviations above average (i.e., 115–130) is rarely seen and a rational is rarely provided in either scenario (e.g., Klein et al., 2004; Desman et al., 2008; Kofler et al., 2010; Mazzone et al., 2011; Antonini et al., 2013). While the implications of having an IQ between 70 and 85 are not clear, reasons for excluding participants with an IQ lower than 70 on the other hand are more established. Below this level (i.e., 70) there may be significant cognitive and adaptive impairments (e.g., Harris, 2013) that may lead to floor effects and outliers in studies examining performance on cognitive and behavioral tasks.

On the other hand, it is important to recognize that depending on the cognitive or behavioral tasks at hand an IQ cut-off of 80 or 85 may be reasonable given the complexity of the task. If this is the case, however, the rationale should be provided by the researchers. This form of rationale is suitably discussed when the researcher is interpreting the results (i.e., Discussion section). This is because the IQ cut-off may be influential in the interpretation of the studies outcomes in addition to addressing limitations. For example, in a study where the tasks are relatively demanding and the IQ cut-off needs to be higher it can be explained in this section of the research paper that the results may not apply to individuals with ADHD who have lower IQ’s. In addition to providing a rationale the limitations that may result from the IQ cut-off, such as limited generalizability, need to be addressed in the Discussion section of the research paper. Unfortunately, this is not common practice.

A meta-analytic examination of differences in full scale IQ among adults with and without ADHD by Bridgett and Walker (2006) addresses the scope of the problem of excluding individuals with ADHD who have lower IQ’s. Among the 18 studies under scrutiny only one explicitly states that they include participants with ADHD with IQ’s 70 and above. Each other used a cut-off of 80 or above or did not take IQ into account. For instance, among the 18 studies under scrutiny that did not explicitly state an IQ cut-off point the mean range of IQ among individuals with ADHD reported in the studies is from 102 to 110. Given that lower IQ is associated with ADHD this suggests that individuals with ADHD may be inaccurately represented.

Similarly, matching participants with and without ADHD on IQ is a common practice (Woods et al., 2002). However, this, in turn, may make any decrements on intelligence tests go unnoticed among individuals with ADHD (Bridgett and Walker, 2006). Likewise, Bridgett and Walker (2006) cite Barkley (1997b, 1998) who note that there is research that recurrently use IQ as a covariate when examining differences between those with and without ADHD in cognitive domains including executive functioning. Barkley (1997b, 1998) makes the arguments that if ADHD and IQ are negatively related then controlling for IQ differences could then eliminate variance that is a result of ADHD in the measures under scrutiny. This course of action may lead to the false conclusion that individuals with and without ADHD perform similarly on a certain measure when in actuality differences are present. What follows is a more detailed explanation of the outcomes associated with IQ exclusionary criteria when ADHD is under study.

## Outcomes

As highlighted, individuals with ADHD score on average 9 points lower on standardized measures of IQ (Frazier et al., 2004). Thus, among people scoring one to two standard deviations lower than the average population in IQ those with ADHD would be over represented in this group. Given individuals with ADHD are more likely to have lower IQ’s researchers that exclude participants with lower IQ fail to take into account the cognitive diversity that exists among this population; limiting the generalizability of the findings by researchers. In turn this may lead to harmful consequences of treatment decisions, an inaccurate understanding of ADHD and a misunderstanding of the relationship between ADHD and IQ.

The detrimental impact of excluding those with lower IQ can be demonstrated by a number of studies. One example, centers on prescribing Atomoxetine, a non-stimulant selective norepinephrine transporter inhibitor. Atomoxetine shows to be effective across gender, ADHD subtypes and age (Michelson et al., 2001, 2002; Wernicke et al., 2001). Further, Sumner et al. (2009) demonstrates that Atomoxetine reduced ADHD symptoms and improves reading scores among children with ADHD and dyslexia. However, their sample excludes participants with IQ’s below 80 and Mazzone et al. (2011) later reported that a lower IQ (i.e., below 85) is associated with a decreased clinical response to the drug. Despite this research, studies continue to examine the efficacy and safety of Atomoxetine while failing to address the influence of IQ (e.g., Fredriksen et al., 2013).

This scenario is not an isolated one, Klein et al. (2004) studied the effectiveness of methylphenidate, another commonly prescribed psychostimulant drug, but also excludes participants with IQ scores lower than 85 despite previous findings by Aman et al. (2003) that children with ADHD and lower IQ scores responds less well to the medication and are more varied in their responses. In addition to limiting our knowledge of ADHD and stimulant medication, this may negatively affect those with lower IQ’s. For instance, clinicians may unknowingly prescribe a less effective medication when there are more suitable alternatives that exist for this population. Overall, given that ADHD is associated with lower than average IQ it is necessary to evaluate the efficacy of a given medication based on results that can be generalizable to this population as a whole. In addition to potentially prescribing ineffective medication to individuals with ADHD who have lower IQ’s through limiting a research sample to participants with average or above average IQ’s we may come to misunderstand ADHD. This approach may again lead to ineffective treatments/interventions.

Excluding ADHD participants who have lower IQ’s may also prevent the development of our understanding of how ADHD may co-exist with other conditions and/or evolve into secondary conditions. This is best elucidated through discussing the relationship ADHD has with other comorbid disorders. It is understood that ADHD frequently co-exists with learning disabilities (LD’s) and sluggish cognitive tempo (Hartman et al., 2004; Barkley, 2014). Both sluggish cognitive tempo and LD’s are related to cognitive impairments (Mayes et al., 2000; Barkley, 2014). Importantly, when ADHD co-exists with LD’s both learning impairments and inattention are exacerbated (Mayes et al., 2000). Similarly, when one has co-existing sluggish cognitive tempo and ADHD the impairments are additive in varied domains of functioning (Barkley, 2012, 2013). Further, conduct disorder and antisocial personality disorder, for example, are common among individuals with ADHD and are associated with lower than average verbal IQ’s (Barkley, 1998; Nigg and Huang-Pollock, 2003). Pure ADHD is uncommon but when it does exist it is associated with higher IQ (Hervey et al., 2004). Thus, excluding ADHD participants who have lower IQ’s may prevent the development of our understanding of how ADHD may evolve into secondary conditions such as antisocial personality disorder or the co-existence of secondary conditions such as LD’s. Thus, researching those with ADHD who have lower IQ may foster an understanding of both their etiology and prevention. Overall, in order for efficacious interventions to take place empirical research needs to include participants with lower IQ’s because of the documented relationship among ADHD and the secondary conditions highlighted.

Taking into account how ADHD and IQ influence one another is also necessary. For instance, the symptoms of ADHD may put individuals with ADHD at an increased risk for lower IQ scores compared to their peers without ADHD (Rommel et al., 2015). Rommel et al. (2015) suggests IQ differences may be a product of failure to benefit from formal education in comparison to their peers without ADHD. Specifically, the common symptoms of ADHD, including inattention, hyperactivity, and impulsivity may limit ability to follow the instructions of teachers and learning from lectures. This may lead to diminishing the benefit education would normally have in the absence of such symptoms. Findings from Washbrook et al. (2013) as well as Scholtens et al. (2013) as cited in the research by Rommel et al. (2015), support this notion; symptoms of ADHD negatively affect academic achievement. Lower education levels in turn are related to reduced performance on Wechsler intelligence subtests (Walker et al., 2009). Furthermore, even more recent research suggests performance on the Raven’s Progressive Matrices test can be explained by education (Sternberg, 2012; Fox and Mitchum, 2013). Thus, Rommel et al. (2015) concludes that education may help explain how the symptoms of ADHD affect IQ scores.

It is also possible that there are subgroups among those with ADHD who are more resilient to the compromising effects ADHD symptoms can have on the skills and knowledge typically acquired through education. For instance, Biederman et al. (2012) finds that individuals with ADHD who do have lower IQ also perform lower on academic measures in comparison to individuals with ADHD who have higher IQ scores. Similarly, the ADHD group with low IQ is more likely to be retained and placed in a special education class. Thus, as opposed to education affecting IQ, IQ could be influencing the benefit of education otherwise received. This is consistent with the finding that those with ADHD and a lower IQ as opposed to those with ADHD with higher IQ’s are more likely to experience social problems and multiple internalizing disorders (e.g., anxiety disorders; Biederman et al., 2012). As such, lower IQ among individuals with ADHD may represent a distinct subgroup that is at greater risk of educational failure and mental health problems. Once again this research points to the necessity of including participants with ADHD who have lower IQ’s in research.

Overall, we have limited understanding of ADHD in low IQ populations as a result of not including those with lower than average IQ in studies. Thus, a significant research gap exists: through failure to study ADHD in low IQ populations (i.e., 70–85) we do not know (1) the base rate of symptoms within this population and (2) whether a distinct diagnostic algorithm would be suitable to those individuals with lower than average IQ who also potentially may have ADHD. Consequently, making inferences about the ADHD population from samples excluding participants with mild intellectual impairments is problematic. It would be remiss not to address the notion that it may be difficult to distinguish between ADHD and behaviors similar to ADHD that occur in response to environmental demands among low IQ populations. However, when a diagnosis of ADHD is made, environmental factors are taken into account. Thus, researchers can be reasonably assured when studying populations with ADHD that the diagnosis is accurate. Next, we make recommendations to help solve the problems that may arise from excluding those with ADHD who have lower IQ’s.

## Recommendations

Dennis et al. (2009) notes that a factor such as IQ cannot be separate from ADHD. Dennis et al. (2009) further explains that it is not uncommon to have matched IQ groups; however, when an ADHD group is matched to a control group with average or above average IQ’s the ADHD group will have higher than average IQ’s than what is the norm for an ADHD sample. As previously highlighted, this may make IQ decrements among individuals with ADHD go unnoticed. We think that including participants with lower IQ’s would address this drawback. At the moment due to the IQ exclusionary criteria, we are problematically focusing our scrutiny on only those with average to above average IQ’s. Thus, it is crucial to select a sample that reflects an accurate representation of the population under study. However, given stringent IQ cut-offs (e.g., >85) we do not think that accurate inferences concerning the ADHD population as a whole are being made. We should aim to make inferences based on the reality of ADHD, not on a manufactured sample with higher than average IQ’s. This approach would help ameliorate the negative outcomes associated with ADHD.

One method is to include participants with mild intellectual impairment but report their results separately. In other words, research samples should be broken down into separate IQ categories (e.g., low IQ range vs. average IQ range), and the results should be reported, respectively. Reporting results in this way would acknowledge the intellectual diversity of the ADHD population and help us capture the differences and similarities that exist between individuals who have ADHD with or without intellectual impairment. Eventually, if the research shows that individuals with average IQ’s (i.e., 85–115) and below average IQ’s but not significantly developmentally delayed (i.e., 70–85) are overwhelmingly similar, then we can simply merge these IQ groups. On the other hand, if differences are observed, then these results would attest to the importance of continuing to examine how IQ and ADHD interact to cause differences in cognitive,behavioral, and emotional outcomes. However, this approach may have drawbacks. Specifically, there may be a reduction in statistical power. Nevertheless in many forms of analyses categorization is inevitable and in turn recruiting large sample sizes is necessary.

One form of an example of this method is through the use of IQ as a moderating variable. For instance, dichotomizing a continuous IQ variable, such that an experimental and control group are subdivided into high and low IQ groups. This approach is taken in de Zeeuw et al. (2012) research investigating the neuroanatomical differences among 214 participants with and without ADHD. This resulted in four subgroups: control participants with low IQ, control participants with high IQ, ADHD participants with low IQ and ADHD participants with high IQ. Through the use of this methodology, de Zeeuw et al. (2012) provides insight into the neuroanatomy of both low and high IQ groups resulting in more precise findings and promoting positive outcomes among individuals with ADHD.

Opposed to an approach where IQ is a moderator or is arbitrarily identified as high or low for the purpose of a given study, acceptable exclusion/inclusion cut-off points will vary depending on the particular issue under investigation. Specifically, there will be different IQ cut-off points for different forms of ADHD studies. For example, in studies where participants need to be able to understand relatively complex tasks researchers may need to put thought into an IQ cut-off. Alternatively, in the event that the tasks involved in a study do not need to be understood we may not need to enforce an IQ cut-off point, such as in studies examining the neuroanatomy of the brain, the genetic basis of ADHD, or studies assessing the efficacy of medications. In such studies, low intellectual functioning is much less likely to interfere with the individual’s full participation in the study. Given that there are many areas of research involving ADHD, rather than propose a specific IQ cut-off point across all studies, we recommend that researchers do not choose an arbitrary cut-off point, but instead justify the cut-offs they choose, and also be open to critiquing others when appropriate justifications are not offered. Over time, consensus will develop over the appropriate cut-offs for each area of research, and their use will become standard practice.

The purpose of this paper is three-fold: (1) to alert researchers to the potential caveats of current exclusionary criteria when studying ADHD, specifically IQ; (2) to have a clear rationale and make note of the limitations given the lack of standards for exclusionary IQ criteria in studies involving participants with ADHD and (3) to provide recommendations to researchers on methods that can be used to include individuals with lower than average IQ’s into studies where ADHD is under scrutiny. Overall, we urge researchers to carefully consider what potential participants they exclude and why they do so. This practice in turn may provide insight into the reality of the complexity of intelligence for clinicians and educators who provide services to clients and/or students with ADHD, leading to more informed diagnoses and treatment in lower IQ cohorts.

## Author Contributions

GM: Came up with the original idea for the paper, wrote the majority of the paper, and found most of the articles used in this mini review. EW: Came up with the idea of adding a recommendations section, thought of the recommendations to offer researchers, and wrote the majority of the recommendations section (including doing the research and finding relevant articles). Contributed to the entire paper through revisions and editing.

## Conflict of Interest Statement

The authors declare that the research was conducted in the absence of any commercial or financial relationships that could be construed as a potential conflict of interest.
